# The complete genome of *Burkholderia phenoliruptrix* strain BR3459a, a symbiont of *Mimosa flocculosa*: highlighting the coexistence of symbiotic and pathogenic genes

**DOI:** 10.1186/1471-2164-15-535

**Published:** 2014-06-28

**Authors:** Luiz Fernando Goda Zuleta, Claúdio de Oliveira Cunha, Fabíola Marques de Carvalho, Luciane Prioli Ciapina, Rangel Celso Souza, Fábio Martins Mercante, Sergio Miana de Faria, José Ivo Baldani, Rosangela Straliotto, Mariangela Hungria, Ana Tereza Ribeiro de Vasconcelos

**Affiliations:** Bioinformatics Laboratory, National Laboratory of Scientific Computation, Av. Getúlio Vargas 333, 25651-075 Petrópolis, RJ Brazil; Department of Biochemistry and Molecular Biology, Federal University of Ceará, 60440-970 Fortaleza, CE Brazil; CNPq, SHIS QI 1, Cj B, Lago Sul, 71605-001 Brasília, DF Brazil; Embrapa Agropecuária Oeste, PO Box 661, 79804-970 Dourados, MS Brazil; Embrapa Agrobiologia, Rodovia BR 465, km 7, 23891-000 Seropédica, RJ Brazil; Embrapa Soja, PO Box 231, 86001-970 Londrina, PR Brazil

**Keywords:** Burkholderia phenoliruptrix, Comparative genomics, Nitrogen-fixing and pathogenic *Burkholderia*

## Abstract

**Background:**

*Burkholderia* species play an important ecological role related to xenobiosis, the promotion of plant growth, the biocontrol of agricultural diseases, and symbiotic and non-symbiotic biological nitrogen fixation. Here, we highlight our study as providing the first complete genome of a symbiotic strain of *B. phenoliruptrix*, BR3459a (=CLA1), which was originally isolated in Brazil from nodules of *Mimosa flocculosa* and is effective in fixing nitrogen in association with this leguminous species.

**Results:**

Genomic comparisons with other pathogenic and non-pathogenic *Burkholderia* strains grouped *B. phenoliruptrix* BR3459a with plant-associated beneficial and environmental species, although it shares a high percentage of its gene repertoire with species of the *B. cepacia* complex (Bcc) and "pseudomallei" group. The genomic analyses showed that the *bce* genes involved in exopolysaccharide production are clustered together in the same genomic region, constituting part of the Group III cluster of non-pathogenic bacteria. Regarding environmental stresses, we highlight genes that might be relevant in responses to osmotic, heat, cold and general stresses. Furthermore, a number of particularly interesting genes involved in the machinery of the T1SS, T2SS, T3SS, T4ASS and T6SS secretion systems were identified. The xenobiotic properties of strain BR3459a were also investigated, and some enzymes involved in the degradation of styrene, nitrotoluene, dioxin, chlorocyclohexane, chlorobenzene and caprolactam were identified. The genomic analyses also revealed a large number of antibiotic-related genes, the most important of which were correlated with streptomycin and novobiocin. The symbiotic plasmid showed high sequence identity with the symbiotic plasmid of *B. phymatum*. Additionally, comparative analysis of 545 housekeeping genes among pathogenic and non-pathogenic *Burkholderia* species strongly supports the definition of a new genus for the second branch, which would include BR3459a.

**Conclusions:**

The analyses of *B. phenoliruptrix* BR3459a showed key property of fixing nitrogen that together with genes for high tolerance to environmental stresses might explain a successful strategy of symbiosis in the tropics. The strain also harbours interesting sets of genes with biotechnological potential. However, the resemblance of certain genes to those of pathogenic *Burkholderia* raise concerns about large-scale applications in agriculture or for bioremediation.

**Electronic supplementary material:**

The online version of this article (doi:10.1186/1471-2164-15-535) contains supplementary material, which is available to authorized users.

## Background

The intriguing biological versatility and the broad range of lifestyles of bacteria belonging to the genus *Burkholderia* have given rise to interest about the genome contents and plasticity of these bacteria with a worldwide distribution [1]. Pathogenic *Burkholderia* have long been studied due to their importance as agents of plant and animal diseases, but special emphasis has been placed on their role as human pathogens [1–3]. However, in the past decade in particular, the increasing number of studies reporting the ecological importance of *Burkholderia* species, showing environmental benefits related to the degradation of xenobiotics, promotion of plant growth, biocontrol of agricultural diseases, and symbiotic and non-symbiotic biological nitrogen fixation, among other characteristics, has renewed interest in these widely adapted bacteria [1, 4–7]. As noted by some authors, obtaining knowledge about *Burkholderia* is a challenging microbiological task, as the genus encompasses strains ranging from those showing excellent agricultural properties to those being capable of devastating the health of cystic fibrosis (CF) patients [8, 9].

Taxonomically, *Pseudomonas cepacia* was originally described by William Burkholder in 1950 as the causative agent of bacterial rot in onion (*Allium cepa*) bulbs [10]; four decades later, seven pathogenic species previously classified as *Pseudomonas* were transferred to the new genus *Burkholderia* [11]. There are currently more than 70 described species within this genus [12]. However, the definition of species within the genus is still one of the most challenging problems in taxonomy, requiring polyphasic approaches [3, 13]. Interest in solving taxonomic problems within the genus has increased in the last decade, as a variety of environmental isolates with great biotechnological potential have been isolated, studied and reported [4, 7, 14, 15]. Unfortunately, there is still no clear distinction to ensure that environmental isolates do not represent a source of human pathogenicity [13]. Therefore, due to our poor knowledge of this genus and inability to distinguish soil and plant-beneficial strains from clinically devastating strains, a reassessment of the risk and a moratorium on the biotechnological applications of *Burkholderia* strains, with an emphasis on the *B. cepacia* complex (Bcc), have been suggested [1, 16, 17].

Symbiotic nitrogen fixation is a key process involved in the global input of nitrogen, and there are now over one and a half centuries of available studies on the nitrogen-fixing bacterial symbionts of legumes (Family Leguminosae, =Fabaceae), collectively referred to as “rhizobia” and classified into the alpha-Proteobacteria class [18]. The first reports, at the turn of this century, of the beta-Proteobacteria symbionts of legumes of the subfamily Mimosoideae were particularly noteworthy, and even more surprising was the identification of these bacteria as *Burkholderia* [19, 20]. Several reports followed these pioneering studies and confirmed that diazotrophic symbiotic *Burkholderia* are broadly dispersed and mainly—but not exclusively—associated with the subfamily Mimosoideae [4–6, 21–24]. Symbiotic *Burkholderia* predominate in the tropics [25, 26], with Brazil and South Africa most likely representing the richest reservoir of these bacteria [4–6, 21, 23].

Analyses of ribosomal and some housekeeping genes of *Burkholderia* species have begun to shed light on evolutionary and taxonomic issues. For example, it has been indicated that typically environmental species might constitute a separate genus [5, 7]. However, a clearer picture will only emerge with the complete genome sequencing of several strains. Particular interest should be paid to the comparative genomics of non-pathogenic and pathogenic strains, but there are unfortunately still very few available genomes of non-pathogenic *Burkholderia*, especially of diazotrophic symbiotic strains.

*Burkholderia phenoliruptrix* is a species that has been reported to accommodate the well-known xenobiotic strain AC1100^T^, capable of degrading 2,4,5-trichlorophenoxyacetic acid [27]. Recently, our group was the first to describe strain BR3459a as a symbiont of *Mimosa flocculosa* [28], while Bournaud and associates [21] were the first to describe *B. phenoliruptrix* as a diazotrophic symbiont of the *Piptadenia* group (Mimosoideae).

Here, we provide a detailed description of the first complete genome of the symbiotic strain of *B. phenoliruptrix* BR3459a (=CLA1), which was originally isolated in Brazil from *M. flocculosa* and is effective in fixing nitrogen in association with this legume. In addition, this strain is outstanding for its capacity to grow *in vitro* at 40°C without losing its symbiotic properties [29].

## Results and discussion

### General genome features

DNA pyrosequencing generated a total of 1,336,569 reads, corresponding to 35-fold genome coverage*,* with 63.15% GC content, 84% of coding region, and an average ORF length of 989 bp. The *B. phenoliruptrix* BR3459a genome consists of two circular chromosomes of 4,152,217 and 2,713,495 bp and one plasmid of 785,419 bp, totalling 7.65 Mbp. The large (chromosome I) and small (chromosome II) chromosomes encode 3,491 and 2,289 predicted ORFs, respectively, while 716 ORFs are present in the plasmid. The chromosome I displays 2,911 known and 580 hypothetical ORFs, 54 tRNAs, 3 rRNA operons, and 7 pseudogenes. The chromosome 2 contains 1,787 ORFs with known function, 502 hypothetical ORFs, 8 tRNAs, 3 rRNA operons, and 7 pseudogenes. In the plasmid, 454 known, 262 hypothetical ORFs, and 15 pseudogenes were identified (Table 1) (http://ligeirinha.lncc.br/bk-final-bin/general_info.cgi).

**Table 1:** **Genomic features of** ***B. phenoliruptrix*** **BR3459a**

Feature	Chromosome 1	Chromosome 2	Plasmid	Total
Size (bp)	4,152,217	2,713,495	785,419	7,651,131
% GC content	63.5	63.7	59.11	-
Number of ORFs	3,491	2,289	716	6,496
Number of known protein ORFs	2,911	1,787	454	5,152
Number of hypothetical ORFs	580	502	262	1,344
Pseudogenes	7	7	15	29
tRNAs	54	8	-	62
rRNAs	9 (3 operons)	9 (3 operons)	-	18

General description of the *Burkholderia phenoliruptrix* BR3459a genome.

### Comparison with other sequenced *Burkholderia*genomes

**Figure 1:**
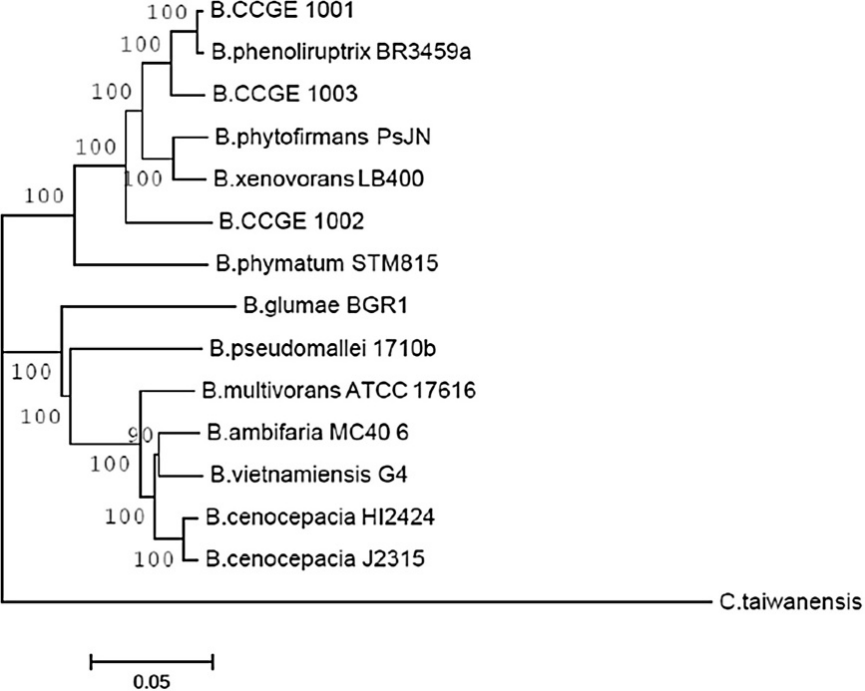
**Concatenated phylogenetic reconstruction of the 545 housekeeping genes based on the Burkholderiales core.** The sequences were aligned and submitted to MEGA5 program, using the neighbour-joining method, with the JTT model. Non-uniformity of evolutionary rates among sites were modelled using a discrete Gamma distribution (+G), with 5 rate categories. All positions showing less than 95% site coverage were eliminated. Bootstrap values are based on 1000 replicates. *Cupriavidus taiwanensis* was used as an outgroup.

For quite some time, studies in *Burkholderia* encompassed only species of clinical interest; however, the recent identification of nitrogen-fixing species has provided an ecological and biotechnological context for the genus [1, 5, 6, 20, 22, 30]. From this perspective, *B. phenoliruptrix* BR3459a was compared with fourteen other *Burkholderia* species, including human and plant pathogens, mutualists and potential agricultural biocontrol agents (Table 2). It has been reported that the *Burkholderia* genus can be phylogenetically distinguished into, at least, two distinct main branches, one of which is composed of pathogens associated with the *B. cepacia* complex (Bcc), and the other of non-pathogenic species associated with plants or related to bioremediation [7, 31]. Under our phylogenetic approach, a similar topology was observed in the trees inferred from 545 housekeeping genes from the fifteen analysed species (Figure 1, Additional file 1: Table S1), in addition to reconstructions using other genes and species (Additional file 2: Figure S1 A). In these reconstructions, *B. phenoliruptrix* is grouped with the plant-associated beneficial and environmental species branch, which includes almost all of the currently known legume-nodulating species as well as other environmental species, while the other group includes pathogenic species. Previously, based on a tree constructed using only 16S rRNA gene sequences, Gyaneshwar and associates [5] proposed that the first group represents a new genus, for which the name *Caballeronia* was suggested. Noteworthy is that recently a new rhizobial genus—*Neorhizobium*—has been proposed based on the analysis of six protein-coding housekeeping genes [32]. Therefore, our robust tree built using 545 housekeeping genes provides strong support for the proposal of the new genus *Caballeronia*.

**Table 2:** **Characteristics of the genomes of the sequenced and annotated** ***Burkholderia*** **species included in study**

Species and strain	Number of chromosomes	Number of plasmids	Size (Mbp)	CG (%)	Lifestyle
*B. ambifaria* MC40-6	3	1	7.64	66	Biocontrol, human pathogen
*B. cenocepacia* HI2424	3	1	7.70	67	Human pathogen
*B. cenocepacia* J2315	3	1	8.06	67	Human pathogen
*B. glumae* BGR1	2	4	7.28	68	Plant pathogen
*B. multivorans* ATCC 17616	3	1	7.01	67	Human pathogen
*B. phenoliruptrix* BR3459a	2	1	7.62	63	Mutualist
*B. phymatum* STM815	2	2	8.68	63	Mutualist
*B. phytofirman*s PsJN	2	1	8.21	62	Biocontrol
*B. pseudomallei* 1710b	2	_	7.31	68	Human pathogen
*B. vietnamiensis* G4	3	5	8.39	67	Mutualist, human pathogen
*B. xenovorans* LB400	3	_	9.73	62	Mutualist
*Burkholderia* sp. CCGE1001	2	_	6.83	64	Mutualist
*Burkholderia* sp. CCGE1002	3	1	7.88	63	Mutualist
*Burkholderia* sp. CCGE1003	2	_	7.05	63	Mutualist
*Cupriavidus taiwanensi*s LMG 19424	2	1	6.48	65	Mutualist

Characteristics of the genome of *Burkholderia phenoliruptrix* BR3459a and other analysed *Burkholderia* species.

**Figure 2:**
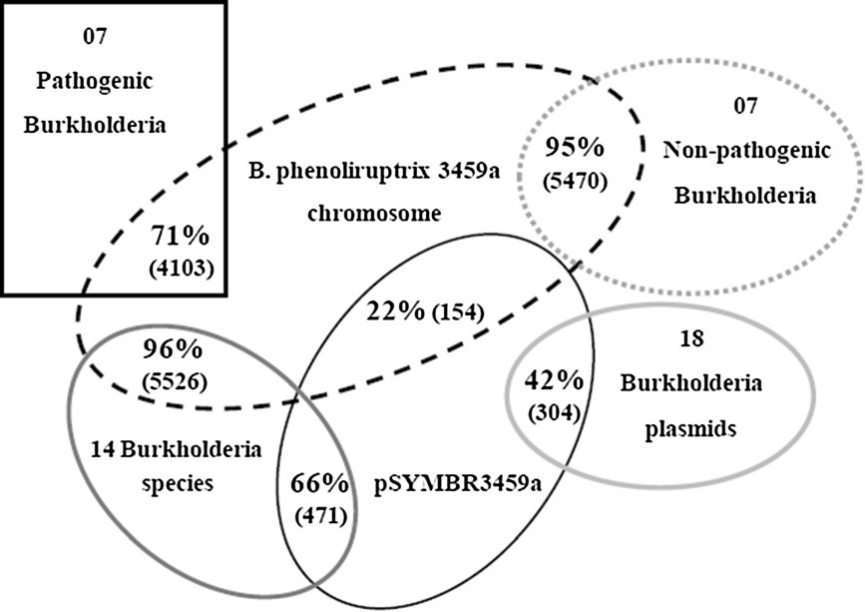
**Venn diagram of the proteins shared between** ***B. phenoliruptrix*** **and other** ***Burkholderia*** **species by distinct Bidirectional Best-Hit comparisons.** Common proteins encoded by genes present in the chromosome and plasmid of *B. phenoliruptrix* in comparisons with chromosomes and plasmids of the fifteen *Burkholderia* species; only the plasmidial sequences; chromosomes of non-pathogenic strains; and chromosomes of pathogenic strains.

We also performed genomic comparisons of *B. phenoliruptrix* using Bidirectional Best-Hit (BBH) methodology among (1) the chromosomes and plasmids of all *Burkholderia* species; (2) all of the plasmids studied; (3) the chromosomes of non-pathogenic strains; and (4) the chromosomes of pathogenic species of interest (Table 2) (Figure 2). The data obtained when the fifteen species were compared showed that the *B. phenoliruptrix* genome is most closely related to the non-pathogenic *Burkholderia* sp. strains CCGE1001 and CCGE1003 and to *B. phytofirmans* PsJN. *Burkholderia* sp. CCGE 1001 was isolated from a nodule of *Mimosa affinis* (subfamily Minosoideae, tribe Mimoseae) in Veracruz, Mexico; while CCGE 1003 was isolated from *Chamaecrista absus* (subfamily Caesalpinioideae, tribe Cassieae) in Tequila, Jalisco, México (Dr. E. A. Ormeño-Orrilo, personal communication); even though both strains lacked the symbiotic genes. In this comparison, we observed that *B. phenoliruptrix* shared 4,929; 4,513 and 4,266 common clusters with *Burkholderia* sp. strains CCGE1001 and CCGE1003 and with *B. phytofirmans* PsJN, respectively. However, 4% and 34% of the *B. phenoliruptrix* coding genes present in the chromosome and plasmid, respectively, did not cluster with any other *Burkholderia* sequences. Of these 82 and 103 proteins not present similarity with other the other species used as comparison. Interestingly, the genomic comparison between the plasmid pSYMBR3459a and the chromosome of *B. phenoliruptrix* showed 154 common clusters, most of which were not derived from duplication processes. This is more representative than the number of proteins shared among pSYMBR3459a and the other *Burkholderia* plasmids, with the exception of the *B. phymatum* pBHY02 plasmid, which presented 199 shared clusters. Within the total pSYMBR3459a repertoire of coding genes, 227 proteins were identified only in this plasmid.

In comparison with pathogenic species, 71% of the proteins encoded by genes in the *B. phenoliruptrix* chromosome were also found in the Bcc complex, a higher percentage than that observed for *B. pseudomallei* 1710b (65%). This divergence could be explained by the unusually large number of simple sequence repeats observed in *B. pseudomallei*, resulting in frameshift, missense, deletion and insertion mutations [33], in addition to a high recombination frequency due to horizontal gene transfer (HGT) events [34].

**Figure 3:**
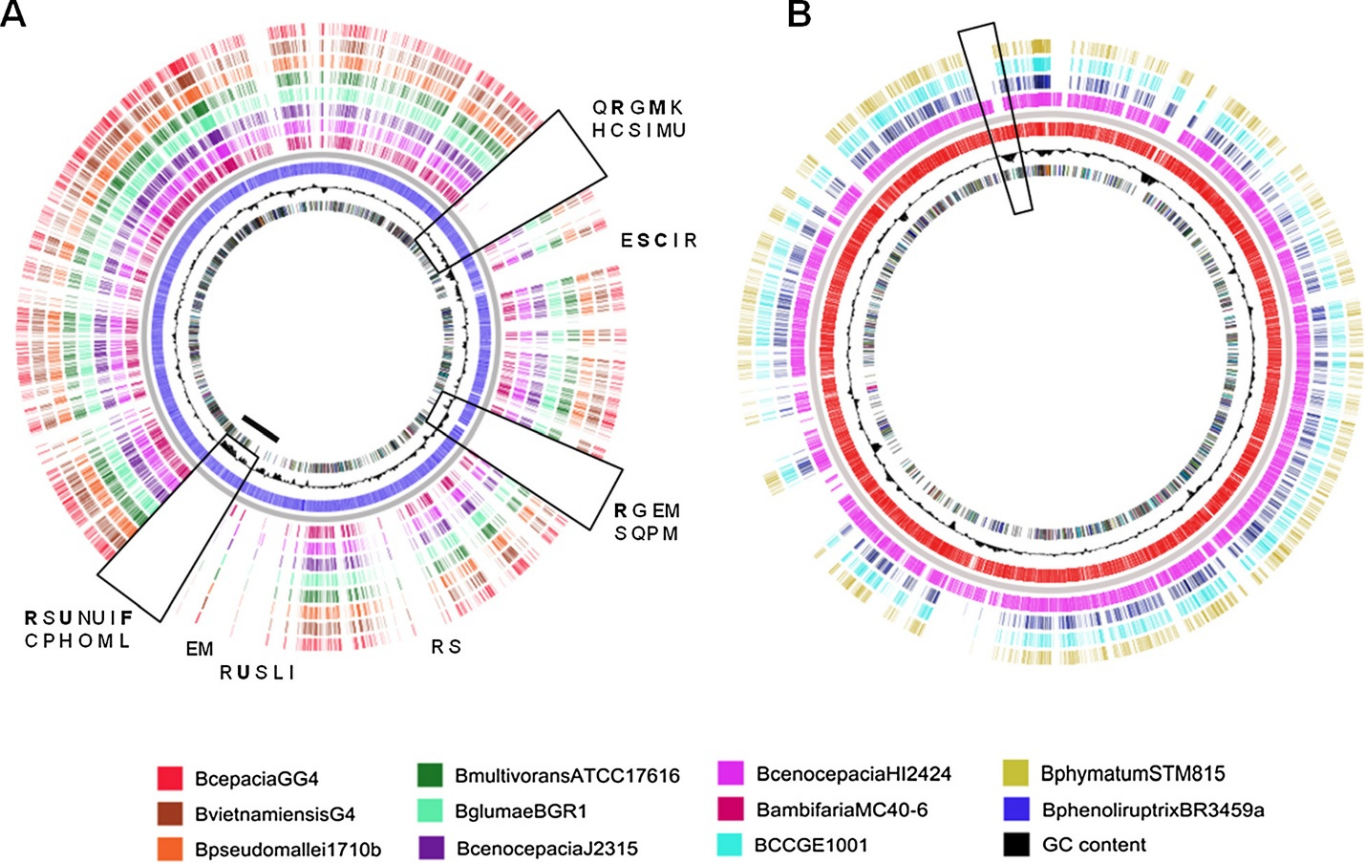
**BLAST Atlas comparison among** ***B. phenoluriptrix*** **BR3459a and pathogenic** ***Burkholderia*** **strains.** The BLAST Atlas comparison among: **(A)** *B. phenoluriptrix* BR3459a and pathogenic *Burkholderia* strains performed by Gview. The dissimilarities regions are highlighted, in addition to the most representative COG categories; **(B)** *B. phenoluriptrix* BR3459a and pathogenic and symbiotic *Burkholderia* species. In highlighted, the region containing a transposase identified in *B. phenoliruptrix*.

The high clustering observed between *B. phenoliruptrix* and Bcc complex species was investigated by additional bidirectional best hit, synteny, and BLAST atlas comparisons. The BLAST Atlas showed that the *B. phenoliruptrix* chromosome presents a large similarity with the pathogenic *Burkholderia*, except in three major regions, which are present only in *B. phenoliruptrix*. One of these regions showed a prevalence of genes with general function prediction only, according to COG. Interesting, this region contains an elevated GC content compared with the remaining chromosome (Figure 3A). A similar map was obtained with *B. cepacia*, the pathogen *B. cenocepacia* HI2424 and the symbionts B. CCGE1001, which were the most similar species to *B. phenoliruptrix* in the BBH analyses, in addition to *B.phymatum* STM815. In *B. phenoliruptrix*, it was found a transposase with 86,21% of identity to *B. cepacia* in a region with distinct GC content (Figure 3B). The main differences are associated with a variation of GC content, which may be indicative of a gene transfer event.

The BBH showed that the pathogen *B. cepacia* GG4 contains 3147 common clusters with *B. phenoliruptrix*, some of which are composed by surface antigens, chemotaxis proteins, and an elevated number of lipoproteins, translocases and proteins involved with flagellar biosynthesis. In comparison, *B. cepacia* GG4 and the symbiotic bacteria *B. phymatum* STM815 share 3024 clusters. Similar clustering was observed in both BBH; however *B. phenoliruptrix* showed a considerable number of proteins associated with the type IV and type VI secretion systems, in addition to pilus assembly and transposase, which were not observed in *B. phymatum* (Additional file 3: Table S2 and Additional file 4: Table S3).

**Figure 4:**
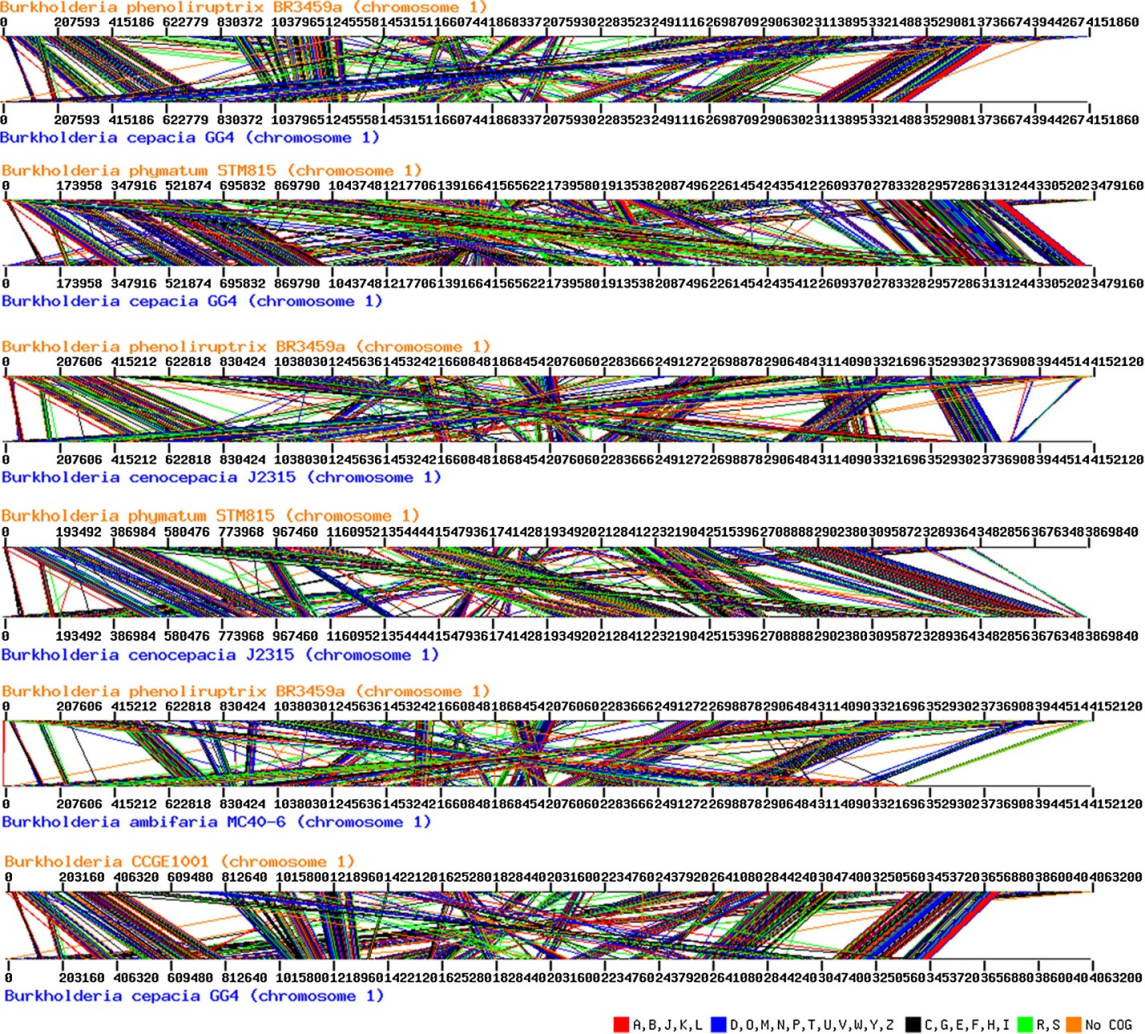
**Syntenies among** ***B. phenoluriptrix*** **BR3459a and pathogenic** ***Burkholderia*** **in comparison with other symbionts strains.** The syntenies among *B. phenoluriptrix* BR3459a and pathogenic *Burkholderia* strains were performed by Perl script based on BBH data. The data were corroborated with the addition of the pathogen *B. cepacia* and the symbionts *B. phymatum* and *B.* CCGE1001 in the comparisons. The syntenic regions were grouped by COG categories.

The synteny data showed that *B. phenoliruptrix* BR3459a presents a considerable gene order in four major regions compared with the pathogen *B. cepacia* GG4, which are more conservative than the observed to *B. cenocepacia* and *B. ambifaria* MC40-6. *B. phymatum* STM815 is more syntenic to *B. cepacia*, although of the lower number of clusters identified, in comparison with *B. phenoliruptrix*. Similar synteny was observed with the pathogen *B. cenocepacia* J2315 (Figure 4)*.* However, *B.phymatum* is not phylogenetically close to BR3459a. The similarity between *B. phenoliruptrix* and the pathogenic *Burkholderia* strains indicates that there is a need for further studies considering the potential use of non-pathogenic *Burkholderia* strains in agriculture and bioremediation.

In the non-pathogenic *Burkholderia* species comparison, the number of shared proteins corresponded to 95% of the *B. phenoliruptrix* genomic repertoire. The vast majority of the 108 genes found only in *B. phenoliruptrix*encoded for hypothetical proteins.

The metabolic pathways identified in *B. phenoliruptrix* BR3459a were compared with those of pathogenic and non-pathogenic species according to KEGG (accessed on October 25^th^, 2013). In general, *B. phenoliruptrix* showed reduced number of genes associated with environmental information processing, translation and metabolism categories. Although of minor magnitude, the reduction was more evident in metabolic pathways, including those related to lysine, valine, leucine and isoleucine degradation (amino acid metabolism); amino sugar and nucleotide sugar metabolism, galactose metabolism, glycolysis/gluconeogenesis, citrate (TCA) cycle, propanoate, pyruvate and butanoate metabolism (carbohydrate metabolism); oxidative phosphorylation (energy metabolism); cyanoamino acid metabolism and beta-alanine metabolism (metabolism of other amino acids); and geraniol, limonene and pinene degradation (metabolism of terpenoids and polyketides) (Additional file 5: Table S4).

### EPS

First we should mention that the original strain BR3459 showed phenotypic polymorphism, with two types of colonies, small non-mucoid and large mucoid colonies, with BR3459a belonging to this second group. Noteworthy was that even though both mucoid and non-mucoid variants were able nodulate *M. flocculosa*, those induced by the non-mucoid variants were ineffective in fixing nitrogen and inferior in promoting root growth (Additional file 6: Figure S2, Additional file 7: Table S5). No EPS (extracellular polysaccharides, or exopolysaccharides) production was observed in the parental BR 3459 and non-mucoid variants (EPS could not be precipitated with cetrimide or ethanol from bacteria grown in yeast-mannitol medium); in addition, the strains could not grow in minimum medium and were less tolerant of high temperatures. Contarily, EPS production, growth in minimum medium and heat tolerance were confirmed in BR3459a and other EPS-producing variants. We have then analyzed with more detail EPS genes in the genome of BR3459a.

EPS are high-molecular weight sugar-based polymers that play a role in bacterial adaptation to different stress conditions, acting as a barrier to harmful compounds and being involved in the establishment of symbiotic and pathogenic relationships with hosts [34]. EPS production has been studied in the *Burkholderia* genus, and at least seven different types of EPS have been identified and their structures determined. Some strains produce a single type of EPS, while others produce mixtures [35, 36]. The most common EPS produced by *Burkholderia* is cepacian, composed of a branched acetylated heptasaccharide repeat unit containing D-glucose, D-rhamnose, D-mannose, D-galactose, and D-glucuronic acid in a ratio of 1:1:1:3:1, which has been identified in different species with different lifestyles [37–39].

Genes involved in cepacian biosynthesis are located within the *bce-I* and *bce-II* gene clusters [39]. These two clusters are present in almost all sequenced *Burkholderia* genomes, and phylogenetic studies have revealed three distinct groups. Groups I and II include clinical and environmental isolates from the *B. cepacia* complex as well as animal and plant pathogenic non-*Bcc* isolates; in all of these bacteria, the two clusters are located apart from each other. Group III harbours non-pathogenic rhizosphere and plant-associated strains in which the *bce-I* and *bce-II* gene clusters are located together. In *B. phenoliruptrix* BR3459a, the *bce* genes are clustered together in the same genomic region, thus constituting part of the group III cluster. This attribute could be related to the division of pathogenic and non-pathogenic strains.

The biosynthesis of the EPS cepacian starts with the formation of activated sugar-nucleotide precursors required for the synthesis of the repeat unit building blocks. Some of the enzymes involved in this step were found in *B. phenoliruptrix* BR3459a, such as BceA (a bifunctional protein with the activities of phosphomannose isomerase and GDP-D-mannose pyrophosphorylase, required for GDP-D-mannose biosynthesis) and BceC (a UDP-glucose dehydrogenase, involved in UDP-glucuronic acid biosynthesis), whose coordinated action with other Bce proteins leads to the production of the nucleotide sugar precursors GDP-D-rhamnose, UDP-D-glucuronic acid and UDP-D-galactose [39].

The synthesis of the activated nucleotide sugar precursors is followed by the assembly of the heptasaccharide repeat unit, catalysed by glycosyltransferase proteins encoded by *bceB*, *bceG*, *bceH*, *bceJ*, *bceK*, and *bceR* [39]. The BceB protein was found in *B. phenoliruptrix* BR3459a, as were some proteins belonging to family 2 and group1 of the glucosyl transferases, which can be associated with this pathway.

The steps after repeat unit assembly involve the polymerisation and export of the EPS to the cell surface through a Wzy-dependent pathway, in which a flippase protein (BceQ) exports the heptasaccharide across the inner membrane and a polysaccharide polymerase (BceI) acts at the periplasmic face of the inner membrane. The BceD, BceE, and BceF proteins are also involved in these steps, and probable coding genes were identified in *B. phenoliruptrix*. The putative BceE protein is a Wza homologue and is therefore predicted to be an outer membrane auxiliary protein that acts as a channel. The activities of BceF as a bacterial tyrosine autokinase and BceD as a phosphotyrosine phosphatase (PTP) protein have been determined, and these two proteins may be involved in regulation of the amount and molecular weight of cepacian [40]. These genes are present in all Bcc and non-Bcc species, with the exception of the *bceM* and *bceU* genes, which are absent from *Burkholderia* species in which the *bce* genes are clustered together, including *B. xenovorans* LB400, *B. phymatum* STM815, *B. phytofirmans* PsJN and *B. graminis* C4D1M. Cepacian may play a role in the survival against desiccation and metal ion stress for some *Burkholderia* strains, thus being advantageous for bacteria to thrive in adverse environments [35]. EPS also influence the stability of soil aggregates through interactions with clay particles, contributing to maintaining the mechanical and physical properties of the soil in which plant roots grow [41]. Finally, we should mention that BR3459a can grow *in vitro* at a high temperature (40°C) [29], and EPS might contribute to this ability.

### Secretion systems

In Gram-negative bacteria, type I secretion is carried out by a translocator composed of three proteins that span the cell envelope. One of these proteins is a specific outer membrane protein (OMP), and the other two are cytoplasmic membrane proteins, consisting of an ATP-binding cassette (ABC) and the so-called membrane fusion or adaptor protein (MFP) [42]. We identified some interesting proteins that could be part of the T1SS machinery, such as weak orthologs of *hlyD* and *hlyB* as well as a complete system formed by *tolC*, *macA* and *macB*, which could be involved in macrolide export.

The type II protein secretion system (T2SS), also known as the secretion-dependent pathway, is a two-step process in which proteins are first translocated across the inner membrane via the Sec or Tat pathway, followed by transport from the periplasm to the exterior via an outer membrane secretin. There are many evidences of relationships between several of the protein constituents of the T2SS, type 4 pilus apparatus and Fla systems of bacteria and archaea [43, 44]. The T2SS can be viewed as consisting of 12 core components: the outer membrane secretin (T2S D); a cytoplasmic ATPase (T2S E); an inner (trans)membrane protein (T2S F); the major (T2S G) and minor (T2S H, I, J, K) pseudopilins, which are facilitators of ATPase attachment to the inner membrane that appear, along with T2S F, to form an inner membrane platform (T2S L, M); the pre-pseudopilin peptidase/methyltransferase (T2S O), which also acts upon type 4 prepilin; and a protein that might be involved in substrate recognition and/or secretin interactions (T2S C). Beyond these core components, there are several other proteins that are not conserved among the majority of genera, including T2S N, and T2S A, T2S B and T2S S [45]. All of these core proteins—with the exception of T2S O and T2S N—were found in *B. phenoliruptrix* BR3459a.

The type III protein secretion system (T3SS) can be found in many Gram-negative bacteria, pathogens and symbionts of animals and plants, mediating elaborate interactions with their hosts. These secretion systems translocate proteins that lack a signal sequence and may require specific chaperones for their secretion. The T3SS export mechanism is usually composed of more than 20 different proteins and includes soluble cytoplasmic proteins, outer membrane proteins, and integral membrane proteins. The T3SS enables bacteria to deliver a variety of effectors directly to the host cytosol through a needle structure, also known as the injectisome, allowing them to manipulate host cellular processes and subvert them for their own benefit [46]. We were unable to find proteins in *B. phenoliruptrix* BR3459a forming the injectisome, such as the needle monomer, inner rod, needle-tip protein, translocators, ruler protein and switch proteins; however, we did identify a number of cytoplasmic proteins, outer membrane proteins, and integral membrane proteins, such as SctC, SctT, SctN, SctL, SctJ, SctU, SctV, HpaP, SctQ, SctR, SctS and SctD. Regarding cellular motility, we found a complete set of ORFs related to the flagellum assembly pathway (Additional file 8: Figure S3).

The type IV protein secretion systems (T4SS) consist of specialised macromolecule delivery machines that are ancestrally related to bacterial conjugation systems. These systems are broadly classified as type IVA (T4ASS) or IVB (T4BSS), depending on whether their structural components resemble the VirB/D4 complex of the plant pathogen *Agrobacterium tumefaciens* or the conjugal transfer system of the self-transmissible IncI plasmid, respectively [47]. Both systems deliver proteins termed effectors directly to the host cytosol via a central pore. Prototypical T4ASS are composed of 11 VirB proteins (VirB1-11) and VirD4, while the Dot/Icm T4BSS requires up to 27 proteins for efficient function [48, 49]. In *B. phenoliruptrix* BR3459a, we found two sets of T4ASS, in the plasmid and in the chromosome, including all of the VirB/D4 proteins except for VirB7.

The type V protein secretion system (T5SS), also known as the autotransporter system, is the simplest and most widespread of these specialised secretion pathways. Classical autotransporters are a large and highly diverse family of polypeptides composed of a cleavable signal sequence, an N-terminal passenger domain and a C-terminal translocator. The signal peptide targets the protein to the Sec complex and initiates translocation across the inner membrane. Subsequently, the translocator is integrated into the outer membrane, and the passenger domain is translocated into the extracellular space, where it typically acts as an adhesin, degradative enzyme, or cytotoxin or mediates another virulence-related function [50]. In *B. phenoliruptrix* BR3459a, we did not identify any virulence factors in the genome.

The type VI protein secretion system (T6SS) consists of a multicomponent secretory machine that delivers effector proteins to both prokaryotic and eukaryotic cells in a contact-dependent manner. It is composed of a minimal set of 13 subunits, which are thought to form the core apparatus. There are also many accessory subunits in this system, whose functions are not yet known, though some of them might be needed for the proper assembly or function of the apparatus [51]. In *B. phenoliruptrix* BR3459a, we found 26 proteins belonging to the T6SS, some of which appear to be membrane-associated proteins, while others are related to tailed bacteriophage components, but no function could be inferred via *in silico* analyses for the great majority of these proteins.

### Environmental stress

One outstanding property of *B. phenoliruptrix* BR3459a is its high tolerance to high temperature, both in vitro and, as shown in Additional file 9: Table S6, being capable of maintaining symbiotic properties in temperatures as high as 40°C. The bacterial stress response can be defined as a cascade of alterations in gene expression and protein activity to allow survival under potentially damaging conditions sensed by bacteria. In this response, the cells become stress resistant and/or eliminate the stress agent, and/or mediate the repair of cell injuries [52].

Bacterial growth and survival may depend on the rate at which osmotic pressure varies and on the amplitude and duration of osmotic pressure changes. There are multiple osmoregulatory systems that appear to be functionally redundant among the organisms studied thus far, which ensure optimal metabolic fitness. These systems may involve osmosensory proteins, divided into three groups: osmosensory transporters, the histidine kinase components of two transcriptional regulatory systems and mechanosensitive channels [53].

Some of the best-characterised osmosensory transporters are ProP, BetP, and the OpuABC complex. ProP is a proton symporter that acts as both an osmosensor and an osmoregulator involved in the early bacterial osmoregulatory response. ProP shows broad substrate specificity, transporting proline and glycine betaine with similar affinities, whereas BetP and OpuA are specific to glycine betaine [54]. BetP carries out high-affinity uptake of glycine betaine, and the OpuABC complex is involved in a multicomponent binding protein-dependent transport system for glycine betaine. These proteins can all detect osmotic pressure changes and respond by mediating osmoprotectant uptake without the assistance of other proteins. In *B. phenoliruptrix* BR3459a, we found homologs of ProP, OpuAA and OpuAB.

Regarding histidine kinase components, some of the best-characterised are KdpD and EnvZ. The former is a member of the two-component regulatory system KdpD/KdpE, involved in the regulation of the *kdpFABC* operon, and the latter is a member of the two-component regulatory system EnvZ/OmpR, involved in the regulation of osmoregulation (genes *ompF* and *ompC*). EnvZ phosphorylates OmpR in response to environmental signals. In BR3459a, we found homologs of KdpD, KdpE, KdpA, KdpB, KdpC, and three copies of EnvZ and OmpR.

The high-affinity ATP-driven potassium transport (or KDP) system consists of many components, such as KdpFABC (which catalyses the hydrolysis of ATP coupled to the exchange of hydrogen and potassium ions) as well as the two-component regulatory system KdpD/KdpE, involved in the regulation of the *kdp* operon. We identified homologs of all of these proteins: KdpF, KdpA, KdpB, KdpC, KdpD, and KdpE. However, we did not find homologs of the components of the TrkAGH system, which is another potassium transport system, and we detected only one unrelated protein that was likely involved in potassium uptake. However, several other transporters could participate in potassium transport, such as the ABC transporters. Additional candidates include the mechanosensitive channels MscL and MscS, which allow the non-specific efflux of cytoplasmic solutes and prevent cell lysis, thus participating in the regulation of osmotic pressure changes within the cell. In BR3459a, we found homologs of both MscL and MscS.

Osmotic gradients dissipate within seconds due to water flux via the phospholipid bilayer and aquaporins (from the Latin words: *aqua* = water and *poru*s = passage), which are transmembrane proteins that display a specific three-dimensional structure with a pore that provides a pathway for water permeation across biological membranes [55]. Both structures are involved in osmoregulation and the maintenance of cell turgor during volume expansion in rapidly growing cells, mediating the rapid entry or exit of water in response to abrupt changes in osmolarity. We found a homolog of AqpZ.

The heat shock response (HSR) is defined as the cellular response to a temperature increase. A major component of this response involves the up-regulation of a set of proteins termed heat shock proteins (*hsp*), notably including chaperones, which help proteins to fold, and proteases, which degrade unfolded proteins. These proteins are usually regulated by a single transcription factor, such as the Sigma 32 transcription factor in *E. coli* and heat shock factor (HSF) in eukaryotic cells [56]. A homolog of Sigma 32 was identified.

In the cold-shock response, CspA and its homologs play a key role; these small proteins (65–70 aa in *E. coli*) have been identified in all types of bacteria except for archaea and cyanobacteria. *E. coli* harbours nine paralogs belonging to the *csp* family; CspA, CspB, CspE, CspG, and CspI are cold-inducible, while CspC, CspD, CspF, and CspH are not. Notably, the nucleic acid-binding domain of the CS proteins, termed the cold-shock domain (CSD), is the most evolutionarily conserved nucleic acid-binding domain found within prokaryotes and eukaryotes [57]. In *Burkholderia*, we found only two orthologs when we subjected the sequences of the nine paralogs to BLAST searches, and those showing the lowest similarity were CspH and CspF.

OtsA is essential for the viability of cells at low temperatures and elevated osmotic strengths. This protein catalyses the transfer of glucose from UDP-glucose to glucose-6-phosphate to form alpha,alpha-trehalose 6-phosphate. OtsB removes the phosphate from trehalose 6-phosphate (Tre6P) to produce free trehalose and catalyses the dephosphorylation of glucose-6-phosphate (Glu6P) and 2-deoxyglucose-6-phosphate (2dGlu6P). We found two orthologs in *Burkholderia*, OtsA and OtsB.

The alternative to these targeted responses to specific stresses is more global changes in metabolism and gene expression that confer protection from many types of stress. Generally, these global responses have been identified under conditions of nutrient deprivation, e.g., as cells enter stationary phase [58]. For example, *E. coli* and related bacteria show increased accumulation of RpoS, a specialised sigma factor, to allow cells to become more resistant not only to the stress that they encounter initially, but also to other stressful treatments. This cross-protection phenomenon is typical of general stress responses and contrasts with specific stress responses that only address the consequences of the specific inducing stress [58]. When the RpoS system is induced, the cell is resistant to a wide range of stress and starvation treatments [59, 60]. We could identify a RpoS ortholog present in *B. phenoliruptrix* strain BR3459a.

Altogether, this large repertoire of genes related to stress tolerance might play roles and explain the tolerance of strain BR3459a to high temperature and environmental conditions in the tropics.

### Symbiotic plasmid

Bacteria that are symbiotically associated with legumes and capable of fixing nitrogen have been studied for over one and a half centuries. Until recently, these bacteria were classified exclusively within the alpha-Proteobacteria class, in the genera *Azorhizobium*, *Allorhizobium* (reclassified as *Rhizobium*), *Bradyrhizobium*, *Mesorhizobium*, *Rhizobium*, *Sinorhizobium* (=*Ensifer*), *Devosia*, *Methylobacterium*, *Ochrobactrum*, *Phyllobacterium* [18] and, more recently, *Aminobacter* [61], *Microvirga* [62], and *Neorhizobium* [32]. It was less than 15 years ago that the first Beta-Proteobacteria were recognised as diazotrophic symbionts of legumes [19, 20], and since then there have been several reports of isolates of the genera *Burkholderia* and *Cupriavidus* from nodules of legumes, in general—but not exclusively—of the subfamily Mimosoideae [5, 21–24]. However, despite the increasing interest in diazotrophic symbiotic beta-Proteobacteria, genomic studies of symbiotic genes are still rare, and the present study highly contributes to reveal some unusual properties of these bacteria.

As previously described in the genome announcement of BR3459a, the symbiotic plasmid pSYMBR3459a shows high sequence identity and considerable gene synteny with the symbiotic plasmid pBPHY02 of *B. phymatum* strain STM815^T^, including genes related to nodulation, nitrogen fixation, auxin synthesis, hydrogenase components and ACC deaminase activity [28]. This plasmid appears to have originated from chromosome 2 of *B. phymatum*, as shown by the phylogeny of the *parA, parB* and ACC deaminase genes (data not shown). However, there is apparently a gene resulting from the fusion of *parA* and *parB* that is phylogenetically related to *B. phenoliruptrix*, indicating that an evolutionary process has occurred in both species and not only a transfer event of the symbiotic plasmid.

In the pSYMBR3459a, starting with the nodulation genes, a unique copy of the regulatory *nodD* was found, followed by *nodA* (an N-acyltransferase required for nodulation), *nodB* and *nodC* (involved in the synthesis of the Nod factor), *nodH* (required for the formation of sulfated nod factor), *nodIJ* (an ABC transporter complex involved in the exportation of the nodulation factors), *nodS* (SAM-utilising methyltransferase involved in Nod factor synthesis) and *nodU* (involved in the 6-O-carbamoylation of Nod-factors). As recently described in *Rhizobium tropici*, NodC directs the synthesis of the Nod factor backbone chitin oligosaccharide structure, which is deacetylated, acylated, methylated, carbamoylated and sulfated by the products of *nodB*, *nodA*, *nodS*, *nodU* and *nodH*, respectively, important features to determine more or less specific Nod factors that are then exported by an ABC-type transporter encoded by *nodIJ* [63].

Symbiotic *Burkholderia* species have mainly been found in association with *Mimosa* species, including *B. tuberum*, *B. mimosarum*, *B. phymatum*, *B. nodosa*, *B. sabiae*, *B. symbiotica* and *B. diazotrophica* [21]. Further studies have shown that nodulation by *Burkholderia* could be extended to other legumes belonging to the Caesalpinoideae and Papilionoideae subfamilies, - observed for *B. tuberum* STM678, as well as for CCGE1003, both strains showing high similarity with BR 3459a. Interesting, although BR 3459a can nodulate some legume species, it is highly specific in fixing nitrogen with *M. flocculosa* (Additional file 10: Table S7), what might be related to nodulation genes. It has been proposed that the nature of the Nod-factor acyl group attached by NodA can contribute to the determination of host range in several rhizobial species, such as in *R. tropici* [63], and in our analysis of *nodA* of BR3459a is more related to *B. phymatum*.

Another interesting feature of pSYMBR3459a is *nodW*, which is a member of a two-component regulatory system regulation of the transcription of genes involved in the nodulation process. *nodW* was first described as the two-component regulatory system *nodV/nodW*, localised to the symbiotic island of *Bradyrhizobium diazoefficiens* USDA 110^T^ [64], also present in the *B. japonicum* strains SEMIA 5079 and USDA 6^T^ [65, 66]. *B. japonicum*/*B. diazoefficiens* exhibit soybean as their main host species, and NodV responds to the isoflavonoid genistein and then phosphorylates its cognate regulator NodW, which in turn, may be required to positively regulate the transcription of one or several unknown genes involved in the nodulation of alternative hosts [mung bean (*Vigna radiata*), cowpea (*Vigna unguiculata),* siratro]. *nodW* was subsequently detected in plasmid “c” of the fast-growing rhizobium *R. etli* [67], and candidate orthologs were proposed in other rhizobial species. NodW is a strong candidate to help in the definition of host specificity, and in BR3459a it also shows high similarity to *B. phymatum*.

The *nif/fix* operons involved in nitrogen fixation are also present in pSYMBR3459a, including *nifA* (a key regulatory protein), *nifE* and *nifN* (both may play a role in biosynthesis of the prosthetic group of nitrogenase), *nifQ* (likely involved in molybdenum processing), *nifH* (an iron protein involved in key enzymatic reactions in nitrogen fixation), *nifD* (a molybdenum-iron protein that constitutes part of the nitrogenase complex that catalyses the key enzymatic reactions in nitrogen fixation).

We identified *nif* operon members such as *nifB* (likely involved in the synthesis of the Fe-Mo cofactor), *nifT*, *nifZ*, *nifW* (may protect the nitrogenase Fe-Mo protein from oxidative damage), *nifX* (may play a role in the processing of the iron-molybdenum cofactor), *nifN* (may play a role in the biosynthesis of the prosthetic group of the nitrogenase FeMo cofactor), *nifE* (may play a role in the biosynthesis of the prosthetic group of the nitrogenase FeMo cofactor), *nifH* (a nitrogenase iron protein), *nifA* (involved in the activation of nif operons), *nifV* (a Fe-Mo-cofactor biosynthetic component), *nifD* and *nifK* (part of the nitrogenase complex that catalyses the key enzymatic reactions in nitrogen fixation), and *nifQ* (likely involved in molybdenum processing). Finally, we also identified *fix* operon members such as *fixU*, *fixX*, *fixC, fixA* and *fixB.*

The presence or absence of the main representatives of the nodulation and nitrogen-fixing proteins in *B. phenoliruptrix* was compared with other *Burkholderia* (Table 3). We observed that the presence of *fix* (FixABCX) and *nif* (NifKDH, NifEN, NifB) coding genes was restricted to the species *B. phenoliruptrix*, *B. phymatum*, *Burkholderia* sp. CCGE1002, *B. xenovorans*, *B. vietnamiensis*, and *C. taiwanensis*. Within this group, an exception was found for NifH, which was also partially identified in *B. phytofirmans*. In contrast, NifQ showed a broad distribution in the BBC complex and non-pathogenic groups. Regarding the nodulation operon, NodDABC was only identified in *B. phenoliruptrix*, *B. phymatum*, *Burkholderia* sp. CCGE1002, and *C. taiwanensis* and was not found in *B. xenovorans* and. *B. vietnamiensis* (Table 3)*.*

**Table 3:** **Proteins involved in nodulation and biological nitrogen fixation in** ***Burkholderia phenoliruptrix*** **BR3459a**

	NodA	NodB	NodC	NodD	FixA	FixB	FixC	FixX	NifH	NifD	NifK	NifE	NifN	NifB	NifQ
**Betaproteobacteria**															
*B. ambifaria* MC40-6	-	-	-	-	-	-	-	-	-	-	-	-	-	-	-
*B. cenocepacia* HI2424	-	-	-	-	-	-	-	-	-	-	-	-	-	-	+
*B. cenocepacia* J2315	-	-	-	-	-	-	-	-	-	-	-	-	-	-	+
*B. glumae* BGR1	-	-	-	-	-	-	-	-	-	-	-	-	-	-	-
*B. multivorans* ATCC 17616	-	-	-	-	++?	++?	-	-	-	-	-	-	-	-	+
*B. phenoliruptrix* BR3459a	+	+	+	+	+	+	+	+	+	+	+	+	+	+	+
*B. phymatum* STM815	+	+	+	+	+	+	+	+	++	+	+	+	+	+	+
*B. phytofirman*s PsJN	-	-	-	-	-	-	-	-	+p	-	-	-	-	-	-
*B. pseudomallei* 1710b	-	-	-	-	-	-	-	-	-	-	-	-	-	-	-
*B. vietnamiensis* G4	-	-	-	-	+	+	+	+	+	+	+	+	+	+	+
*B. xenovorans* LB400	-	-	-	-	+	+	+	+	+	+	+	+	+	+	+
*Burkholderia* sp. CCGE1001	-	-	-	-	-	-	-	-	-	-	-	-	-	-	-
*Burkholderia* sp. CCGE1002	+	+	+	+	+	+	+	+	+	+	+	+	+	+	+
*Burkholderia* sp. CCGE1003	-	-	-	-	-	-	-	-	-	-	-	-	-	-	-
*Cupriavidus taiwanensi*s LMG 19424	+	+	+	+	+	+	+	+	+	+	+	+	+	+	+
**Alphaproteobacteria**															
*Azorhizobium caulinodans* ORS 571	+	+	-	+	+	+	+	+	+	+	+	+	+	+	+
*Rhizobium etli* CFN 42 p42d	+	+	+	+	+	+	+	+	+	+	+	+	+	+	+
*Ensifer meliloti* 1021 pSymA	-	+	+	+	+	+	+	+	+	+	+	+	+	+	-
*Bradyrhizobium japonicum* USDA110	+	+	-	+	+	+	+	+	+	+	+	+	+	+	+
*Rhizobium loti* MAFF303099															
*Rhizobium leguminosarum bv viciae* 3841 pRL10	+	+	+	+	+	+	+	+	+	+	+	+	+	+	-
*Bradyrhizobium* BTAi1	-	-	-	-	+	+	+	+	+	+	+	+	+	+	+
*Bradyrhizobium* ORS278	-	-	-	+	+	+	+	+	+	+	+	+	+	+	+
*Xanthobacter autotrophicus* Py2	-	-	-	+	+	+	+	+	+	+	+	+	+	+	+
*Rhizobium* sp NGR234	-	-	-	+	+	+	+	+	+	++	+	+	+	+	+
*Rhodopseudomonas palustris BisA53*	-	-	-	-	+	+	+	+	+	+	+	+	+	+	+
*Agrobacterium tumefaciens* C58	-	-	-	-	-	-	-	-	-	-	-	-	-	-	-
*Aurantimonas* SI85 9A1	-	-	-	-	-	-	-	-	-	-	-	-	-	-	-
*Agrobacterium radiobacter* K84	-	-	-	-	-	-	-	-	-	-	-	-	-	-	-
*Agrobacterium vitis* S4	-	-	-	-	-	-	-	-	-	-	-	-	-	-	-

Presence and absence of the *B. phenoliruptrix* proteins related to nodulation and biological fixation processes in comparison with some Beta- and Alpha-proteobacteria . ++? proteins not identified by BLAST, but present in the Uniprot database (with more than one copy); +p partial sequence identified; ++ two sequences identified for the described protein.

**Figure 5:**
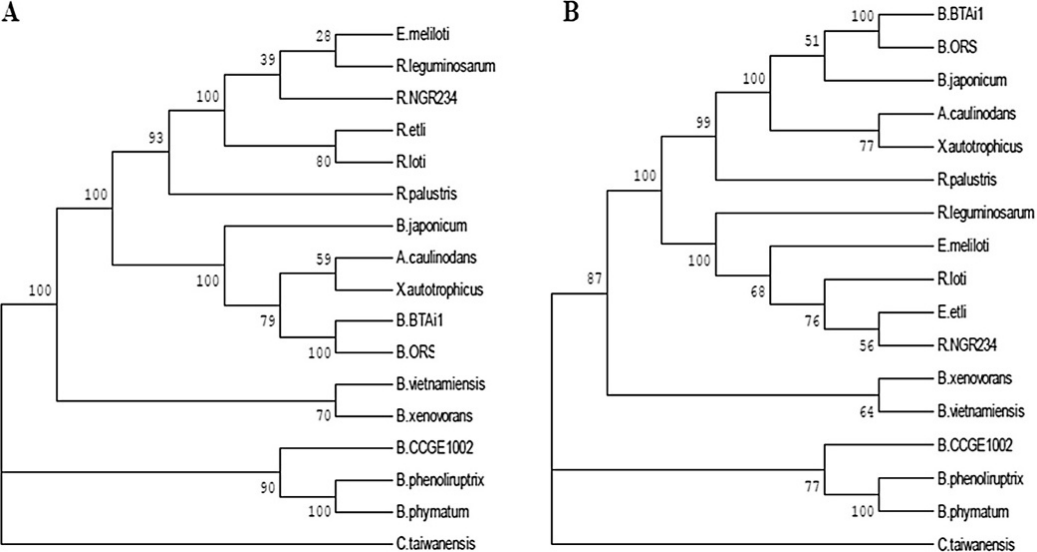
**Phylogenetic trees generated for the NifHDK (A) and NifEN (B) concatenated amino acid sequences from the analysed** ***Burkholderia*** **species and some Rhizobiales species.** The sequences were aligned and submitted to the MEGA5 program, using the maximum likelihood method and WAG + G + I **(A)** and JTT + G **(B)** models, with 5 rate categories. All positions showing less than 95% site coverage were eliminated. Bootstrap values were based on 1000 replicates. *Cupriavidus taiwanensis* was used as outgroup.

**Figure 6:**
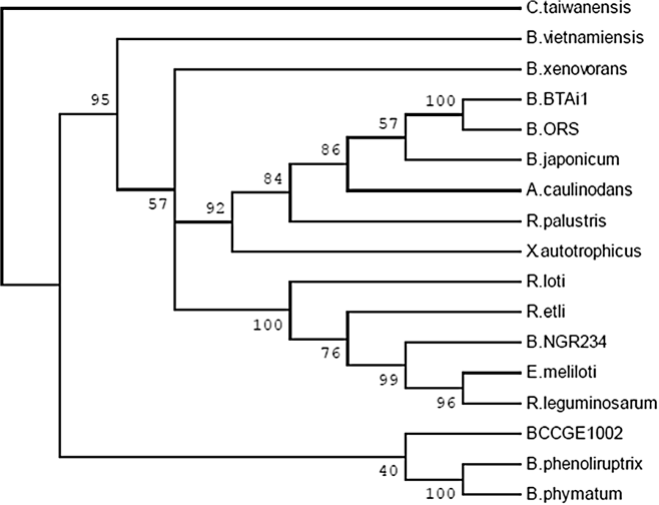
**Phylogenetic tree generated for the FixABC concatenated amino acid sequences proteins from the analysed** ***Burkholderia*** **species and some Rhizobiales species.** The sequences were aligned and submitted to the MEGA5 program, using the maximum likelihood method and WAG + G model, with 5 rate categories. All positions showing less than 95% site coverage were eliminated. Bootstrap values were based on 1000 replicates. *Cupriavidus taiwanensis* was used as an outgroup.

The Fix and Nif phylogenies showed that *B. phenoliruptrix* was closer to *B. phymatum* and *Burkholderia* sp. CCGE1002, and *B. xenovorans* was grouped with *B. vietnamiensis* (Figures 5 and 6). However, in the 16S rRNA tree, *B. phenoliruptrix* is also related to *Burkholderia* sp. CCGE1001, while *B. xenovorans* and *B. phytofirmans* descend from the same ancestor, and *B. vietnamiensis* and *B. multivorans* are grouped on the same branch (Additional file 2: Figure S1 B).

Nodulation is an event with ancient origin that occurs among most *Burkholderia* strains [4]. It can be inferred that the absence of nodulation genes observed in *B. xenovorans* and *B. vietnamiensis* occurred in the ancestor of these species. However, greater representativeness is needed to support this hypothesis.

With respect to nitrogen-fixing genes, gene loss events were also observed in CCGE1001, *B. phytofirmans* and *B. multivorans.* According to Zhu and collaborators [31], events of gene acquisition and reduction appear to represent a trait of *Burkholderia* evolution. In a genomic context, the occurrence of at least one horizontal gene transfer (HGT) event can be observed in the majority gene families in this genus. Furthermore, it has been proposed that gene loss is an event more frequently observed in closely related species [31]. Although HGT can occur in nitrogen-fixing genes, it is not a common event in *Burkholderia*, in contrast to observed in alpha-Proteobacteria rhizobia [4]. However, the determination of nitrogen-fixing or pathogenic phenotypes in *Burkholderia* appears to be influenced by several factors other than gene gain and loss events, as observed by Menard and collaborators [68]. It has been reported that the *nif* cluster is present in approximately 25% of *Burkholderia* species. For example, several *B. vietnamiensis* isolates from cystic fibrosis patients have been shown to express the dinitrogenase complex. However, the disease conditions may have favoured the *nif* deletion and the selection of variants with nitrogen-fixing ability affected [68].

### Xenobiosis

The species *B. phenoliruptrix* is known for the xenobiotic properties of strain AC1100^T^, which is capable of degrading halophenol and 2,4,5-trichlorophenoxyacetic acid (2,4,5-T), a potent herbicide that is a component of Agent Orange [27]. Experiments have shown that this strain is capable of degrading additional halogen-substituted phenol compounds, including 2,4,5-trichlorophenol, 2,4,5,6-tetrachlorophenol and pentachlorophenol [69]. *B. vietnamiensis*, another plant-growth-promoting bacterium, also displays bioremediation properties, being capable of degrading aromatic hydrocarbons such as benzene and toluene. *B. xenovorans* is an environmental organism of economic importance due to its capacity to degrade polychlorinated biphenyl (PCB) compounds [70]. In strain BR3459a, we found that 4% of the putative genes in the plasmid and in the chromosomes are related to xenobiotic metabolism. Significant enzymatic representations were observed for the KEGG categories related to xylene, polycyclic aromatic hydrocarbon, furfural, fluorobenzoate, chloroalkane, chloroalkene, atrazine, benzoate and aminobenzoate degradation. Some enzymes involved to styrene, nitrotoluene, dioxin, chlorocyclohexane, chlorobenzene and caprolactam degradation were identified in *B. phenoliruptrix*.

When we analysed xylene degradation, we identified some interesting proteins involved in catechol formation through anthranilate catabolism or via benzoate degradation. Examples of these proteins include some (AndAa, AndAb) that are part of the multicomponent enzyme anthranilate dioxygenase system, which converts anthranilate to catechol; the complete 2-halobenzoate dioxygenase multicomponent enzyme system, consisting of a heterohexamer formed by *cbdA*, *cbdB,* and *cbdC*, which catalyses the incorporation of both atoms of molecular oxygen into 2-halobenzoate to form catechol; and 1,6-dihydroxycyclohexa-2,4-diene-1-carboxylate dehydrogenase (*benD*), which generates catechol from benzoate. Within this pathway, we also identified *mhpD*, *mhpE* and *mhpF*, which are the final enzymes in the meta-cleavage pathway for the degradation of aromatic compounds. We analysed the other *Burkholderia* strains (Table 2) and observed the complete 2-halobenzoate dioxygenase multicomponent enzyme system in all strains, except for *Burkholderia* sp. CCGE1003 and *B. glumae,* whereas in *B. pseudomallei* 1710b we identified only two components of this system. BenD was not found in *Burkholderia sp*. CCGE1003, *B. glumae* or *B. cenocepacia* J2315. MhpDEF was not identified in *Burkholderia* sp. CCGE1003, *B. cenocepacia* HI2424, *B. ambifaria* MC40-6, *B. cenocepacia* J2315 or *B. multivorans* ATCC 17616, while in *B. glumae* and *B. pseudomallei* 1710b, we did not detect *mhpD*.

Concerning polycyclic aromatic hydrocarbon degradation, we identified both subunits (alpha and beta chains) of protocatechuate 4,5-dioxygenase, which is responsible for the aromatic ring fission of protocatechuate, and both subunits (alpha and beta chains) of protocatechuate 3,4-dioxygenase, which plays an essential role in the utilisation of numerous aromatic and hydroaromatic compounds via the beta-ketoadipate pathway. We analysed the other *Burkholderia* strains (Table 2) and found both subunits of protocatechuate 3,4-dioxygenase in all strains, whereas both subunits of protocatechuate 4,5-dioxygenase were only detected in *Burkholderia sp*. CCGE1002, *Burkholderia sp*. CCGE1003 and *B. phymatum*.

Concerning furfural degradation, almost all of the enzymes involved in this process were present, indicating the possibility that in *B. phenoliruptrix*, furfural is converted to 2-Furoyl-CoA and then to S-(5-Hydroxy-2-furoyl)-CoA by HmfH, HmfD, and HmfABC. The action of HmfH and HmfFG can also result in the formation of 2-Furoate, an intermediate of 2-Furoyl-CoA, from 5-Hydroxymethyl-2-furaldehyde, and in the final step, 2-Oxoglutarate is formed by HmfE and enters the citrate cycle. We analysed the other *Burkholderia* strains (Table 2) and found the same enzymes described above in all of the mutualists strains, whereas none of these enzymes were identified in the pathogenic strains, except for HmfH in *B. cenocepacia*.

Considering fluorobenzoate degradation, we also identified all of the enzymes involved in 3-fluorobenzoate degradation via hydroxylation, forming 2-maleylacetate as a final product, including 1,6-dihydroxycyclohexa-2,4-diene-1-carboxylate dehydrogenase, catechol 1,2-dioxygenase, muconate cycloisomerase, and carboxymethylenebutenolidase, among others. We also identified a 2-halobenzoate dioxygenase multicomponent enzyme system formed by *cbdA*, *cbdB*, and *cbdC* (an NADH:acceptor reductase), which catalyses the incorporation of both atoms of molecular oxygen into 2-halobenzoate to form catechol. We analysed the other *Burkholderia* strains (Table 2) and found the enzymes cited above in all of them, except for *Burkholderia sp*. CCGE1003, *B. glumae* and *B. cenocepacia* J235, whose 3-Fluorobenzoate degradation pathways were incomplete, missing 1,6-dihydroxycyclohexa-2, 4-diene-1-carboxylate dehydrogenase and benzoate 1,2-dioxygenase. Notably, we detected chloromuconate cycloisomerase only in *B. xenovorans*; this enzyme permits an alternative pathway that could be used by this strain to degrade 3-Fluorobenzoate, forming 2-Maleylacetate.

Regarding chloroalkane and chloroalkene degradation, we identified alcohol dehydrogenase, aldehyde dehydrogenase, haloacetate dehalogenase, and 2-haloacid dehalogenase, which are enzymes that convert 2-chloroethanol into glycolate. In this pathway, genes encoding both components of the nitrogenase complex, i.e., the iron protein and the molybdenum-iron protein, which are involved in key enzymatic reactions in nitrogen fixation, are present in *B. phenoliruptrix*. We analysed the other *Burkholderia* strains (Table 2) and found the enzymes involved in the conversion of 2-chloroethanol into glycolate in all of them, except for alcohol dehydrogenase, which was absent in *Burkholderia pseudomallei* 1710b, *B. glumae* and *B. phytofirmans*. With respect to the two components of the nitrogenase complex, we did not find both of them in the majority of the pathogenic strains (*B. pseudomallei* 1710b, *B. multivorans* ATCC 17616, *B. glumae*, *B. ambifaria* MC40-6, *B. cenocepacia* J2315 and *B. cenocepacia* H2424). Additionally, we were unable to identify them in some beneficial species as well (*Burkholderia* sp. CCGE1001, *Burkholderia* sp. CCGE1003 and *B. phytofirmans*).

Considering atrazine degradation, all three subunits (alpha, beta and gamma) associated with the formation of urease, involved in urea degradation, were identified. We analysed the other *Burkholderia* strains (Table 2) and found the same enzymes described above in all of the strains. *B. xenovorans* was the strain in which the most enzymes were identified; thus, this species is capable of converting atrazine or ammelide into cyanuric acid through many different routes.

With respect to benzoate degradation, we identified all of the enzymes responsible for converting benzoate into beta-ketoadipyl-CoA, a precursor of succinyl-CoA that is processed in the TCA cycle. In this pathway, almost all the enzymes required to form 2-oxo-2,3-dihydrofuran-5-acetate (an intermediate product found in the beta-ketoadipate pathway) from 4-hydroxybenzoyl-CoA were identified. The 3,4-dihydroxybenzoate intermediate formed in these steps can also be converted into oxaloacetate and pyruvate by the enzymes protocatechuate 4,5-dioxygenase (subunits alpha and beta), 2-hydroxy-4-carboxymuconate semialdehyde hemiacetal dehydrogenase, 2-pyrone-4,6-dicarboxylate lactonase, 4-oxalmesaconate hydratase and 4-hydroxy-4-methyl-2-oxoglutarate aldolase. Enzymes involved in the production of acetyl-CoA by crotonoyl-CoA and acetaldehyde were also found. In *B. cenocepacia* J2315, *B. glumae* and *Burkholderia sp*. CCGE1003, we were unable to detect the initial steps of benzoate conversion into beta-ketoadipyl-CoA; in these strains, this process most likely begins with catechol. The enzyme 4-hydroxybenzoyl-CoA thioesterase, involved in 2-oxo-2,3-dihydrofuran-5-acetate formation, was not present in any of the pathogenic strains, except in *B. glumae*. The enzymes involved in the conversion of 3,4-dihydroxybenzoate into oxaloacetate and pyruvate could also not be identified in any of the pathogenic strains, except for 1–2 enzymes found in *B. glumae* and *B. multivorans* ATCC 17616. However, these enzymes were also absent in some of the symbiotic strains, such as in *Burkholderia sp*. CCGE1001, *B. xenovorans* and *B. phytofirmans*. The enzymes involved in the production of acetyl-CoA by crotonoyl-CoA were found in all strains. However, acetaldehyde dehydrogenase was absent in *B. ambifaria* MC40-6, *B. cenocepacia* HI2424, *B. multivorans* ATCC 17616 and *Burkholderia sp*. CCGE1003.

When we analysed the aminobenzoate degradation category, we identified almost all of the subunits of the multicomponent enzyme toluene-4-monooxygenase (T4MO), which hydroxylates toluene to form p-cresol. We also detected the toluene-4-sulfonate monooxygenase system iron-sulphur subunits TsaM1 and TsaM2, involved in the toluene-4-sulfonate degradation pathway. With the protocatechuate degradation pathway, we identified the protocatechuate 4,5-dioxygenase alpha and beta chains, which are responsible for the aromatic ring fission of protocatechuate, and 2-pyrone-4,6-dicarbaxylate lactonase, which is involved in the meta-fission degradative pathway for protocatechuate via catalysing the reversible hydrolysis of 2-pyrone-4,6-dicarboxylate (PDC) to a mixture of 4-carboxy-2-hydroxymuconate (CHM) and 4-oxalomesaconate (OMA). A flavoprotein, an iron-sulphur protein, cytochrome b-556 and a hydrophobic protein were also detected, constituting the multicomponent enzyme succinate dehydrogenase (SDH), which is related to carbohydrate metabolism, being involved in the formation of fumarate from succinate. Furthermore, we identified some of the subunits of the multicomponent enzyme toluene-4-monooxygenase (T4MO) in *Burkholderia sp*. CCGE1002, *B. phymatum*, *B. xenovorans*, *B. phytofirmans*, *B. glumae*, *B. multivorans* and *B. pseudomallei*. Regarding the degradative pathway for protocatechuate, we detected the protocatechuate 4,5-dioxygenase alpha and beta chains in *Burkholderia sp*. CCGE1002, *Burkholderia sp*. CCGE1003 and *B. phymatum*. The enzyme 2-pyrone-4,6-dicarbaxylate lactonase was present only in *B. phymatum*.

### Antibiotic resistance

*Burkholderia* species are unusual not only due to being among the most antimicrobial-resistant microorganisms identified to date, showing resistance to several types of antibiotics, but also because of their unique ability to use one of these antibiotics, penicillin, as a nutrient [8]. The genomic analysis of strain BR3459a revealed a large number of antibiotic-related genes. Among these genes, the ones that encode to streptomycin and novobiocin are noteworthy. We found two copies of AmrA, AmrB and OprA in *B. phenoliruptrix* BR3459a. These proteins compose a multidrug efflux system operon, which has been shown to be responsible for aminoglycoside and macrolide resistance in *B. pseudomallei* [71].

When we analysed the streptomycin pathway from the KEGG database, we found only few of the relevant genes in strain BR3459a. In this pathway, D-glucose-6P is converted into two different products in a series of enzyme-catalysed reactions, forming streptidine-6P and dTDP-L-dihydro-streptose. Only the first three steps required for streptidine-6P formation were observed in this strain. The final enzyme in this process, dTDP-dihydrostreptose-streptidine-6-phosphate dihydrostreptosyltransferase (EC = 2.4.2.27), was absent as well. We analysed the other mutualistic and pathogenic *Burkholderia* strains (Table 2) and found the same few genes described above in all of them.

Regarding the novobiocin pathway, only the enzymes involved in the initial steps of converting prephenate or L-tyrosine into 4-hydroxyphenylpyruvate were identified. We analysed the other mutualistic and pathogenic *Burkholderia* strains (Table 2) and found the same several genes described above in all of them, except for aspartate aminotransferase, which was identified only in *B. phenoliruptrix*. We were also unable to detect prephenate dehydrogenase in *B. pseudomallei*.

## Conclusions

*Burkholderia* is an intriguing and important genus encompassing a variety of species and strains, ranging from highly pathogenic organisms to strains that promote plant growth. In this manuscript, we describe the complete genome of *B. phenoliruptrix* BR3459a, a nitrogen-fixing, heat-tolerant strain isolated from a nodule of *M. flocculosa* in Brazil. It has been suggested that *Burkholderia* species can be split in two main branches, one of which is composed of pathogens associated with the Bcc complex, while the other consists of non-pathogenic species associated with plants or related to bioremediation [7, 31]. In this study, a robust phylogenetic tree built using 545 housekeeping genes provided strong support for the definition of a new genus for the second branch, which would include BR3459a. Fortunately, there are increasing evidences that bacteria within the second branch would not be pathogenic, e.g. it has been recently demonstrated, by bioinformatics and functional tests, that the risk of opportunistic infection by symbiotic *Burkholderia* is extremely low [72]. However, we should be cautious, as we showed that 71% of the proteins encoded by the chromosomal genes of BR3459a had correspondence with the Bcc complex and "*pseudomallei*" group. The symbiotic plasmid of BR3459a showed high sequence identity with the symbiotic plasmid of *B. phymatum*. In the genome, interesting genes were identified in BR3459a, including genes related to EPS synthesis, to different types of secretion systems and to stress tolerance. An outstanding repertoire of genes with biotechnological potential was revealed, with an emphasis on xenobiotics, showing that, in addition to the key property of nitrogen fixation, BR3459a could show other applications, such as in bioremediation.

## Methods

Bacterial strain *Burkholderia phenoliruptrix* strain BR3459a (=CLA1) is described as a mucoid colony variant of the heat-tolerant β-rhizobium strain BR3459 (=BR3462) [29]. The strain was isolated from *M. flocculosa* Burkart (bracatinga-de-campo-mourão)*,* of subfamily Mimosoideae, tribe Mimoseae, which is a small tree species endemic to southern South America [29]. In a typical medium for rhizobial growth—yeast-mannitol broth [73]—the strain is characterised by fast growth rate and acid reaction.

### Genome sequencing, assembly and functional annotation

Standard protocols were employed to extract DNA, and library generation and sequencing were performed at the Darcy Fontoura de Almeida Computational Genomics Unit (UGCDFA) of the National Laboratory of Scientific Computation (LNCC) (Petrópolis, RJ, Brazil). Two sequencing libraries were prepared following GS FLX Titanium series protocols: one Rapid Library and one Paired End Library ¨C 3 kb span, using 500 ng and 5 μg of genomic DNA, respectively. The titration emulsion PCR and sequencing steps were carried out according to the manufacturer's protocol. A two region 454 sequencing run was performed on a 70×75 PicoTiterPlate (PTP) using the FLX Genome Sequencer System (Roche Diagnostics Corporation), one region was loaded with the Rapid Library and another one with the 3 kb Paired End Library.

The sequences were assembled using both Newbler (version 2.6) (454 Life Sciences, Roche Diagnostics Corporation, Branford, CT) and the Celera Assembler (WGS, version 7.0). The results of the two assemblies were aligned to each other with the program cross match (package Phred/Phrap/Consed), as they may be complementary, which is effective for closing gaps. Each assembly was mapped against the following reference genomes: *Burkholderia* sp. CCGE1001, *Burkholderia* sp. CCGE1002, *Burkholderia* sp. CCGE1003 and *B. phymatum* STM815. The scaffolds sequences generated by Newbler showed a higher identity with *B*. sp. CCGE1001 and all scaffolds are collinear with this strain. Based on the assembly generated by Newbler, the gaps within and between scaffolds were closed using sequences enclosed in the contigs generated by the Celera Assembler that were identified by the program cross match. The remaining gaps were closed using a mini assembly strategy and the structure (ordering and orientation) of all scaffolds were confirmed by these mini assemblies. Briefly, the assembly was performed using only reads belonging to each pair of contigs selected from a scaffold or by mapping short contigs (<2000 pb) not contained into the scaffolds onto the reference genome. In the chromosome 1, there are 3 scaffolds separated by 3 rRNA operon copies. The chromosome 2 presents 5 scaffolds separated by 3 rRNA operon copies and 2 copies of duplicated IS66 transposases. The junction of each scaffold pair was confirmed by each of mini assemblies. The remaining scaffold corresponds to the plasmid. All of the consensus sequences of each new contig and the sequence that closed each gap were aligned and joined using the program Consed (version 20.0).

The annotation and analysis of the sequences were carried out using the System for Automated Bacterial Integrated Annotation (SABIA) [74]. Automatic functional annotation was conducted using the KEGG database, according to the follow criteria: *i)* ORFs with a good BlastP coverage in the KEGG database, with a minimum of 50% positive identity, 60% query coverage, 80% subject coverage, and 10^-5^ e-value were assigned as “valid”. The first three hits were analysed, and the product was imported from KEGG ORTHOLOGY (KO), if there was one associated with the hit, or KEGG GENES definition, if no KO was associated with the first three hits. *ii)* ORFs that displayed (1) no BlastP hits in the following databases: NCBI-nr, KEGG, UniProtKB/Swiss-Prot, TCDB and Interpro, and (2) the first three BlastP hits product on KEGG containing the keyword hypothetical were assigned as “hypothetical”.

Manual annotation based on comparison with the UniProt/Swiss-Prot, KEGG, NCBI-NR and InterPro databases was performed for ORFs that did not fit the above criteria.

### Comparative genomics

The chromosomal and plasmidial sequences of 15 species of pathogenic and non-pathogenic *Burkholderia* species: *B. ambifaria* MC40-6, *B. cenocepacia* HI2424, *B. cenocepacia* J2315, *B. glumae* BGR1, *B. multivorans* ATCC 17616, *B. phenoliruptrix* BR3459a, *B. phymatum* STM815, *B. phytofirmans* PsJN, *B. pseudomallei* 1710b, *B. vietnamiensis* G4, *B. xenovorans* LB400, *Burkholderia sp*. CCGE1001, *Burkholderia sp.* CCGE1002, *Burkholderia sp*. CCGE1003 and *Cupriavidus taiwanensis* LMG 19424, were obtained by the FTP protocol from GenBank (NCBI) and used in genomic comparisons by the Bidirectional Best-Hits (BBH) clustering method [75]. This method compares the genome of each species against each of the other genomes using the BLAST program [76] to identify pairs of corresponding genes (clusters) and to recognise the best "hit" in other genomes. The genomic comparisons were performed among (1) the chromosomes and plasmids of the fifteen *Burkholderia* species; (2) only the plasmidial sequences; (3) the chromosomes of non-pathogenic strains; and (4) chromosomes of pathogenic strains. Additional BBH comparisons were done between *B. cepacia* GG4 and *B. phenoliruptrix* BR3459a or *B.phymatum* STM815. The parameters applied were a genome coverage and similarity of 60% and a minimum e-value 10^-5^.

The syntenies were obatined using a Perl script applied to BBH data. The BLAST Atlas analyses were performed by Gview program [77].

For phylogenetic reconstructions, 16SrRNA and the nucleotide and amino acid sequences were obtained from the RDP database [78], from GenBank and UniProt databanks. The sequences were aligned with T-coffee [79]. The maximum likelihood method in the MEGA5 program [80] and the model determined by Test Model were applied.

To obtain a coding housekeeping gene phylogeny, the Burkholderiales core genes [81] were recovered into the clusters generated by comparative analysis among the fifteen *Burkholderia* species examined in study. Among the 649 genes present in the dataset, 114 displayed paralogs in the sampled species and were eliminated. The 545 remaining genes (Additional file 1: Table S1) were concatenated, and alignment was performed using ClustalX [82]. The reconstruction was generated with MEGA5 using the neighbour-joining method and JTT + G model. In all reconstructions, positions showing less than 95% site coverage were removed. The bootstrap values were based on 1,000 replicates.

### Supporting data

The complete genome sequence of *Burkholderia phenoliruptrix* BR3459a was deposited at NCBI GenBank under accession numbers CP003863, CP003864, and CP003865, as previously described [28]. The assembly and annotation data are available at http://ligeirinha.lncc.br/bk-final-bin/general_info.cgi.

The sequences used for phylogenetic reconstructions and comparative analysis are available at supporting data section into http://ligeirinha.lncc.br/bk-final-bin/general_info.cgi, in addition to the nucleic acid and protein sequences and atomic coordinates of the genome.

## Electronic supplementary material

Additional file 1: Table S1: The 545 housekeeping genes from *B. cenocepacia* J2315 used as a reference dataset for the phylogeny of the Burkholderiales core. The housekeeping dataset used as a reference for the *Burkholderia* phylogeny based on the Burkholderiales core. The core was identified in the best-bidirectional clusters obtained in the genomic comparisons, and the paralogs were removed from the dataset, resulting in 545 coding genes. (XLS 86 KB)

Additional file 2: Figure S1: Phylogenetic reconstruction based on 16S rRNA, *rec*A, *gyr*B and *atp*D sequences from *Burkholderia* species and the phylogenetic tree generated from the 16S rRNA sequences of the fifteen *Burkholderia* species included in this study. **(A)** Phylogenetic reconstruction based on 16S rRNA, *rec*A, *gyr*B and *atp*D sequences from some *Burkholderia* species. The sequences were aligned and submitted to the MEGA5 program, using the maximum likelihood method, with the TN93 model. Non-uniformity of evolutionary rates among sites may be modelled using a discrete Gamma distribution (+G) with 5 rate categories and by assuming that a certain fraction of sites are evolutionarily invariable (+I). The analysis involved 33 nucleotide sequences. The mutualistic and pathogenic species groups examined in this study are shown (G). **(B)** 16S rRNA phylogenetic tree generated from the sequences of the fifteen *Burkholderia* species included in this study. The sequences were aligned and submitted to the MEGA5 program, using the maximum likelihood method and the GTR + G + I model with 5 rate categories. The mutualistic and pathogenic species included in this study are shown in black and grey, respectively. In both phylogenies, all positions with less than 95% site coverage were eliminated. Bootstrap values are based on 1000 replicates. *Cupriavidus taiwanensis* was used as an outgroup. (TIFF 198 KB)

Additional file 3: Table S2: Comparison of *Burkholderia cepacia* GG4 and *B. phenoliruptrix* BR3459a by BBH. Description of the clusters in common between *B. phenoliruptrix* BR3459a (BUPH) and *B. cepacia* GG4 (GEM) identified by Bidirectional Best Hit comparison. (XLS 786 KB)

Additional file 4: Table S3: Comparison of *Burkholderia cepacia* GG4 and *B. phymatum* STM815 by BBH. Description of the clusters in common between *B. cepacia* GG4 (GEM) and *B.phymatum* STM815 (Bphy) identified by Bidirectional Best Hit comparison. (XLS 722 KB)

Additional file 5: Table S4: Comparison of *Burkholderia* metabolic pathways by KEGG. *B. phenoliruptrix* BR3459a metabolic pathways in comparison with those of the pathogenic and non-pathogenic species analysed, according to KEGG (accessed on October 25^th^, 2013). (XLS 2 MB)

Additional file 6: Figure S2: Response of *Mimosa flocculosa* plants to seed inoculation with rhizobial strain BR3459 variants. *Mimosa flocculosa* plants were inoculated with rhizobial strain BR3459 and its mucoid (BR3459a, BR3459c and BR3459d) or non-mucoid (BR3459b, BR3459e and BR3459f) colony variants. Plants were harvested 90 days after germination. (PNG 563 KB)

Additional file 7: Table S5: Response of *Mimosa flocculosa* plants to seed inoculation with mucoid or non-mucoid colony variants of rhizobial strain BR3459. Plants were grown in N-free stetile substrate and were harvested 90 days after germination. Values are means of three independents cultures and three repetions per culture. NS^1^: no significant differences. (XLS 27 KB)

Additional file 8: Figure S3: Representation of the genes associated with the flagellar assembly pathway. Identified in *Burkholderia phenoliruptrix* 3459a by KEGG. (TIFF 228 KB)

Additional file 9: Table S6: Response of *Mimosa flocculosa* plants to seed inoculation with rhizobial strain BR3459 and its mucoid or non-mucoid colony variants. 1 Bacteria were grown at 40°C in aerated liquid YM medium. Plants were grown in a N-free sterile substrate and were harvested 90 days after germination. The presented values are the means of three repetitions. Additional treatments were as follows: BR3459 grown at 30°C; a mixture of the six BR3459 variants (BR3459Mix:a-f), grown at 40°C and mixed in equal parts just prior to seed inoculation; plants treated with combined nitrogen (N-CONTROL received a total of 140 mg N plant-1); and plants grown in the absence of a N-source (CONTROL). 2 NS: no significant differences. 3 For each parameter, means with the same letters are not significantly different by Duncan's multiple range test (p ≤ 0.05). 4 For each parameter, means with the same letters are not significantly different by the Scott-Knott test (p ≤ 0.05). (XLS 31 KB)

Additional file 10: Table S7: Response of 38 leguminous species to seed inoculation with rhizobial strain BR3459a Nod^-^, absence of nodulation, no root nodule-like structures were induced by bacteria; Fix^-^, bacteria induced the formation of ineffective (non-nitrogen-fixing) nodules; Fix^+^, bacteria induced the formation of effective (nitrogen-fixing) nodules. ^1^Seeds were supplied by Embrapa Agrobiology (RJ/Brazil), and plants were grown in Leonard Jars under greenhouse conditions at Embrapa Agrobiology. Plants were harvested 60 days after germination, with five replications. ^2^Seeds were supplied by the National Botanic Garden of Belgium (Meise/Belgium), and plants were grown under growth chamber conditions at the F.A. Janssens Laboratory of Genetics (Catholic University of Leuven, Belgium). Plants were grown in sterile glass tubes (3.0 x 20.0 cm) under a 12/12 hr light/dark cycle, at 28/26°C, with 60% RH and a photosynthetic photon flux density of 200 μE m^-2^ s^-1^. The substrate was a sand:vermiculite (1:2, v/v) mixture supplied with modified Norris solution every four days. Plants were harvested 45 days after germination, with five replications. ^3^Seeds were supplied by Embrapa Agrobiology (RJ/Brazil), and plants were grown in Leuven, as described above. 4 Cultivars tested: *Phaseolus lunatus* (cvs. gr. Potato and gr. Sieva); *Phaseolus vulgaris* (cvs.Carioca, Carioca-80, Aporé, Pérola, IPA-7, Negro Argel, Rio Tibagi and var. Aborigenes [wild type]); *Glycine max* (cvs. Doko and BRS-285) and *Vigna unguiculata*. (XLS 29 KB)
