# Supplementary figures and images for: The complete genome of Burkholderia phenoliruptrix strain BR3459a, a symbiont of Mimosa flocculosa: highlighting the coexistence of symbiotic and pathogenic genes
Source: BMC Genomics. 2014 Jun 28;15(1):535. doi: 10.1186/1471-2164-15-535 (PMC4101177; doi:10.1186/1471-2164-15-535)

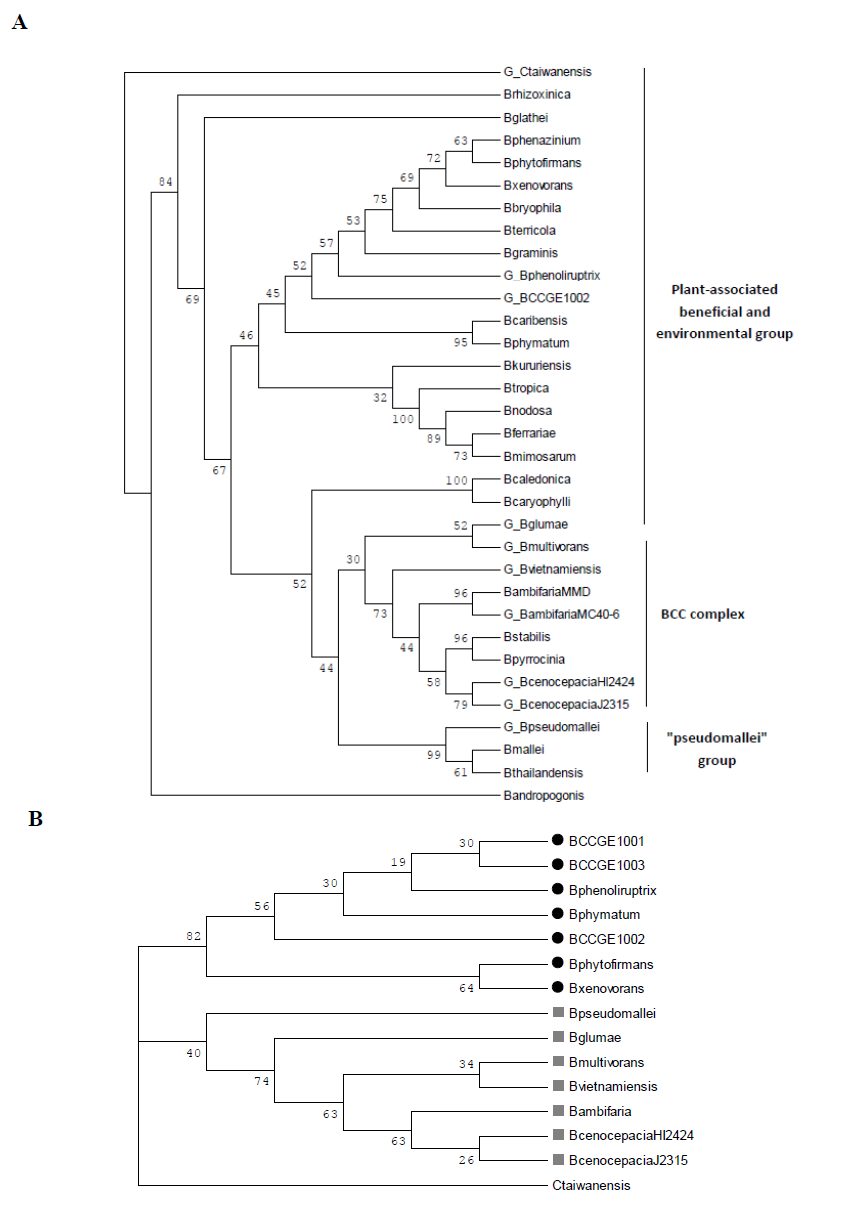

Supplement: Supplementary file 2 — Additional file 2: Figure S1: Phylogenetic reconstruction based on 16S rRNA, recA, gyrB and atpD sequences from Burkholderia species and the phylogenetic tree generated from the 16S rRNA sequences of the fifteen Burkholderia species included in this study. (A) Phylogenetic reconstruction based on 16S rRNA, recA, gyrB and atpD sequences from some Burkholderia species. The sequences were aligned and submitted to the MEGA5 program, using the maximum likelihood method, with the TN93 model. Non-uniformity of evolutionary rates among sites may be modelled using a discrete Gamma distribution (+G) with 5 rate categories and by assuming that a certain fraction of sites are evolutionarily invariable (+I). The analysis involved 33 nucleotide sequences. The mutualistic and pathogenic species groups examined in this study are shown (G). (B) 16S rRNA phylogenetic tree generated from the sequences of the fifteen Burkholderia species included in this study. The sequences were aligned and submitted to the MEGA5 program, using the maximum likelihood method and the GTR + G + I model with 5 rate categories. The mutualistic and pathogenic species included in this study are shown in black and grey, respectively. In both phylogenies, all positions with less than 95% site coverage were eliminated. Bootstrap values are based on 1000 replicates. Cupriavidus taiwanensis was used as an outgroup. (TIFF 198 KB) [file 12864_2014_6246_MOESM2_ESM.tiff]

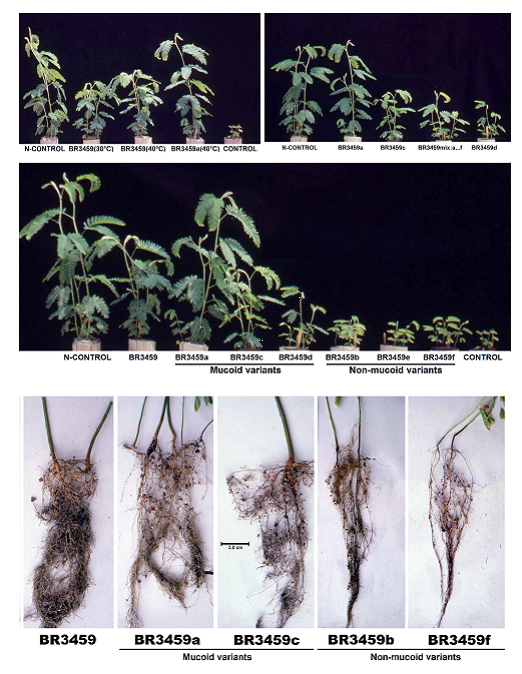

Supplement: Supplementary file 6 — Additional file 6: Figure S2: Response of Mimosa flocculosa plants to seed inoculation with rhizobial strain BR3459 variants. Mimosa flocculosa plants were inoculated with rhizobial strain BR3459 and its mucoid (BR3459a, BR3459c and BR3459d) or non-mucoid (BR3459b, BR3459e and BR3459f) colony variants. Plants were harvested 90 days after germination. (PNG 563 KB) [file 12864_2014_6246_MOESM6_ESM.png]

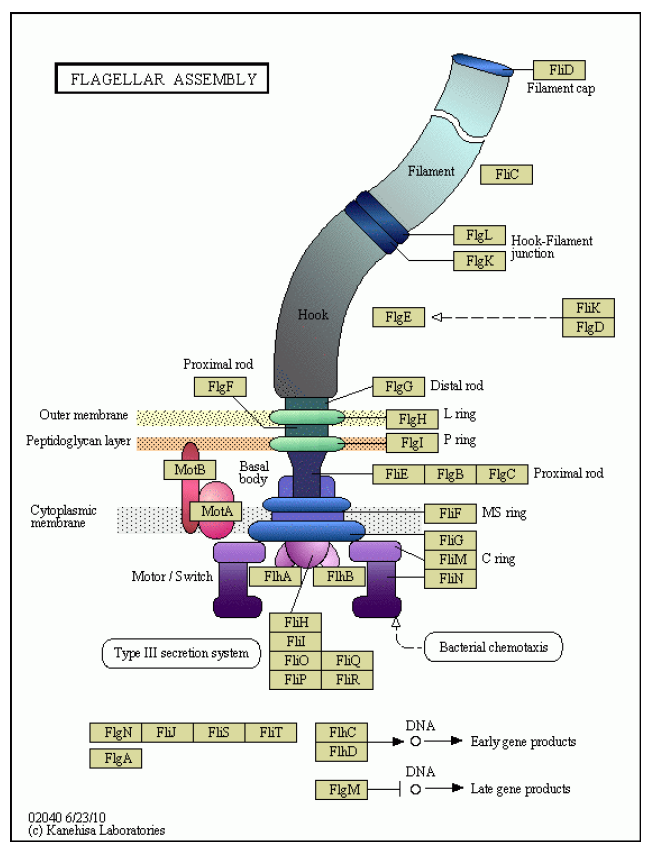

Supplement: Supplementary file 8 — Additional file 8: Figure S3: Representation of the genes associated with the flagellar assembly pathway. Identified in Burkholderia phenoliruptrix 3459a by KEGG. (TIFF 228 KB) [file 12864_2014_6246_MOESM8_ESM.tiff]
